# Supplementary material for: Evaluation of SLC6A8 species conservation and the effect of pathogenic variants on creatine transport
Source: HGG Adv. 2025 Aug 7;6(4):100489. doi: 10.1016/j.xhgg.2025.100489 (PMC12398244; doi:10.1016/j.xhgg.2025.100489)
Supplement: Document S1. Table S1 [file mmc1.pdf]

HGGA, Volume *6*

Supplemental information

Evaluation of *SLC6A8* species conservation and the effect of pathogenic variants on creatine transport

Taryn Diep and Gerald S. Lipshutz

Supplementary Table 1

Cell Line	Mutation	Race	Age at First Symptom	Age at Diagnosis	Human Phenotype		Treatment	Notes
SD1	c.1429-1596DEL	Asian	1 year	2.5 years	Global developmental delay<br>Fine motor delay<br>Regression of fine motor skills<br>Delayed social development<br>Abnormal social behavior<br>Short stature<br>Decreased free carnitine<br>Decreased cerebral creatine	HP:0001263<br>HP:0010862<br>HP:0033692<br>HP:0007101<br>HP:0000735<br>HP:0004322<br>HP:0012250<br>HP:0012447	Psychological therapy	Mother screened for elevated TSH and was on thyroid replacement therapy during pregnancy.<br>Mosaic mutation present in 10.38% of cells
SD2	p.F408DEL	White	6 months	1 year	Abnormality of neuronal electrophysiology<br>Encephalopathy<br>Focal-onset seizure<br>Hypotonia<br>Sleep disturbance<br>Delayed speech and language development<br>Global developmental delay<br>Gross motor delay<br>Constipation<br>Feeding difficulties<br>Autism Spectrum Disorder	HP:0012638<br>HP:0001298<br>HP:0007359<br>HP:0001252<br>HP:0002360<br>HP:0000750<br>HP:0001263<br>HP:0002194<br>HP:0002019<br>HP:0011968<br>HP:0000729	N/A	N/A
SD3	p.L276_V280 DUP	White	Birth	10 years	Failure to thrive<br>Global developmental delay<br>Ataxia<br>Autism Spectrum Disorder<br>Intellectual disability<br>Absent speech<br>Abnormal sensory processing<br>Aggressive behavior	HP:0001508<br>HP:0001263<br>HP:0001251<br>HP:0000729<br>HP:0001249<br>HP:0001344<br>HP:0011443<br>HP:0000718	Communication or learning devices; Levetiracetam (Keppra), creatine guconate, arginine base powder, Lorazepam (Ativan).	Family history of hearing problems, learning difficulties, mild depression, and anxiety
SD4	p.W566*	White	1 year	3 years	Global developmental delay<br>Autism Spectrum Disorder	HP:0001263<br>HP:0000729	Speech therapy and communication or learning devices<br>Guanfacine 2ug/day.	MR spectroscopy detected a low creatine peak, high creatine excretion, cerebral creatine deficiency, and increased urinary creatine/creatinine ratio<br>Unaffected carrier mother
SD5	p.P382L	N/A	Under 3 years	6 years	Global developmental delay<br>Failure to thrive<br>Behavioral abnormality<br>Autism Spectrum Disorder<br>Reduced sensitivity to pain<br>Hyperactivity<br>Dystonia<br>Dyskinesia<br>Delayed speech and language development	HP:0001263<br>HP:0001508<br>HP:0000708<br>HP:0000729<br>HP:0007328<br>HP:0000752<br>HP:0001332<br>HP:0100623<br>HP:0000750	N/A	N/A
SD6	c.912+36_913-18DEL Insertion + Stop Codon	N/A	N/A	N/A	N/A	N/A	N/A	Anonymized sample;<br>data unknown
SD7	p.G67D	White	18 months	34 months	Childhood apraxia of speech<br>Delayed speech and language development	HP:0025194<br>HP:0000750	Speech therapy; Arginine, Glycine, Creatine.	EEG was normal, magnetic resonance spectroscopy showed low peak of creatine in brain parenchyma
SD8	p.F107DEL	N/A	2 years	3 months	Febrile seizures<br>Seizure<br>Status epilepticus<br>Focal-onset seizure<br>Drug-resistant epilepsy<br>Behavioral abnormality<br>Intellectual disability	HP:0002373<br>HP:0001250<br>HP:0002133<br>HP:0007359<br>HP:0002128<br>HP:0000708<br>HP:0001249	Stereotactic EEG and right middle frontal gyrus focal resection<br>Glycerol phenylbutrate at 11.2 mL/m2/day	N/A
SD9	p.P544L	White			Psychomotor delay<br>Severe speech and language delay<br>Growth delay<br>Moderate intellectual disability<br>Attention deficit hyperactivity disorder<br>Oral motor apraxia<br>Muscle hypotrophy	HP:0000753<br>HP:0012758<br>HP:0001510<br>HP:0002342<br>HP:0007018<br>HP:0002427<br>HP:0003199	Valproate (no data), methylphenidate	Mother presented with mild learning disabilities, family history negative for developmental or language delay

Supplementary Materials and Methods

Analysis of SLC6A8 Sequences

The accession numbers for the CLUSTALW alignment were: *Homo sapiens* AAH12355.1, *Nomascus leucogenys* (Northern white-cheeked gibbon, an ape species) XP_030662305.1, *Pongo abelii* (Sumatran orangutan) XP_024096173.1, *Gorilla gorilla gorilla* (Western lowland gorilla) XP_030861991.1, *Macaca nemestrina* (Southern pig-tailed macaque, an Old-World monkey) XP_011715137.1, *Pan troglodytes* (Chimpanzee) XP_016803162.2, *Saimiri boliviensis boliviensis* (Black-capped squirrel monkey, a New-World monkey) XP_039320087.1, *Mus musculus* (House mouse) NP_001136281.1, *Sciurus carolinensis* (Eastern gray squirrel) XP_047392696.1, *Rattus norvegicus* (Brown rat) NP_059044.1, *Canis lupus familiaris* (Dog) XP_025316409.1, *Felis catus* (Cat) XP_023105266.2, *Marmota flaviventris* (Yellow-bellied marmot) XP_027777545.1, and *Danio rerio* (zebrafish) NC_133183.1.
