# Supplementary material for: Evaluation of SLC6A8 species conservation and the effect of pathogenic variants on creatine transport
Source: HGG Adv. 2025 Aug 7;6(4):100489. doi: 10.1016/j.xhgg.2025.100489 (PMC12398244; doi:10.1016/j.xhgg.2025.100489)
Supplement: Document S2. Article plus supplemental information [file mmc2.pdf]

HGG Advances

BRIEF COMMUNICATION

Evaluation of *SLC6A8* species conservation and the effect of pathogenic variants on creatine transport

Taryn Diep[1] and Gerald S. Lipshutz[1,2,3,4,5,6,7,*]

Summary

Creatine phosphate is a high-energy molecule essential for the normal functioning of highly metabolically active organs and tissues. *SLC6A8* encodes the only known creatine transporter in humans (CRT1); pathogenic variants result in a neurophenotype that includes intellectual disability, seizures, and autistic-like behaviors. Due to the importance of creatine phosphate in normal brain function, we compared the amino acid sequence among a group of terrestrial mammals and zebrafish. Finding high interspecies invariance, we (1) sought to quantitatively assess the effect of a number of existing disease-causing *SLC6A8* variants on *in vitro* creatine uptake, comparing variant type/location, along with (2) the reported effect of missense variants on severity classification. Creatine uptake in the pathogenic variants studied demonstrated that the vast majority had a profound effect on uptake; only 1, in a peripheral extracellular loop, had a moderately reduced effect. Of the missense variant analysis, those occurring in C and N termini were tolerated more, while variants in transmembrane domains tended to more likely affect function. While the high degree of amino acid conservation across terrestrial mammals underscores its evolutionary importance, together with the variant analysis, these findings provide a framework for understanding genotype-phenotype correlations in variants of CRT1 and highlight the critical functional constraints.

Introduction

Creatine transporter (CRT1) deficiency (CTD, MIM: 300352), due to loss of function variants in solute carrier family 6 member A8 (*SLC6A8*), is thought to be the second most common class of X-linked intellectual disabilities,[1] and likely is underdiagnosed.[2] The syndrome, which can vary in clinical manifestation, includes hallmark features of intellectual and growth delays, severe speech-language delay, and autistic features[2]; it often includes seizures, behavioral issues, and motor dysfunction.[3,4] Decreased muscle mass and gastrointestinal abnormalities are common.[5] Lack of brain creatine is a key magnetic resonance spectroscopy (MRS) finding.[6] The disorder affects all males and about 50% of female carriers, who typically demonstrate milder intellectual, learning, or cognitive disabilities. The current standard of care is creatine supplementation with arginine and glycine,[7] and while follow-up may show muscular benefits in some, in general, this regimen does not result in improvement in cognitive or psychiatric manifestations or brain MRS creatine levels.[8]

CRT1 co-transports creatine across the cell membrane against a large concentration gradient.[7,9] As 1 of the 19 human sodium-dependent family of SLC6 transporters, it consists of 635 amino acids as 12 transmembrane (TM) spanning α-helices flanked with small N- and C-terminal cytosolic domains. *SLC6A8* is a member of the LeuT fold family of membrane sodium-coupled transport proteins,[10] with key features including a substrate binding site in the center, ion-coupled transport, and an alternating access mechanism with conformational change for substrate translocation.[11] The intracellular and extracellular loops connecting TM domains are thought to be involved in binding and regulation of transport, while the N- and C-terminal cytoplasmic domains are believed to be regions important in trafficking.[12] Mutations may occur in any region and can affect transporter function by (1) impairing creatine binding, (2) preventing conformational changes needed for transport, (3) misfolding with endoplasmic reticulum retention/reduced trafficking, (4) truncation, (5) frameshift resulting in loss of function, or (6) splice-site mutations with aberrant mRNA processing/exon skipping.[13]

SLC6A8 is highly expressed in organs/tissues with high energy demand—brain, heart, kidney and skeletal muscle—resulting in abundant intracellular creatine, important in energy storage and release.[14] Phosphocreatine serves as an intracellular energy reservoir by donating its phosphate group to ADP, allowing for rapid ATP regeneration.[15] While creatine biosynthesis does occur, in the brain this is limited being insufficient to meet its high energy demand.

[1]Department of Surgery, David Geffen School of Medicine at University of California, Los Angeles (UCLA), Los Angeles, CA 90095, USA; [2]Molecular Biology Institute, David Geffen School of Medicine at UCLA, Los Angeles, CA 90095, USA; [3]Departments of Molecular and Medical Pharmacology, David Geffen School of Medicine at UCLA, Los Angeles, CA 90095, USA; [4]Department of Psychiatry, David Geffen School of Medicine at UCLA, Los Angeles, CA 90095, USA; [5]Intellectual and Developmental Disabilities Research Center at UCLA, David Geffen School of Medicine at UCLA, Los Angeles, CA 90095, USA; [6]Semel Institute for Neuroscience, David Geffen School of Medicine at UCLA, Los Angeles, CA 90095, USA
[7]Lead contact
*Correspondence: glipshutz@mednet.ucla.edu
https://doi.org/10.1016/j.xhgg.2025.100489.
© 2025 The Authors. Published by Elsevier Inc. on behalf of American Society of Human Genetics.
This is an open access article under the CC BY license (http://creativecommons.org/licenses/by/4.0/).

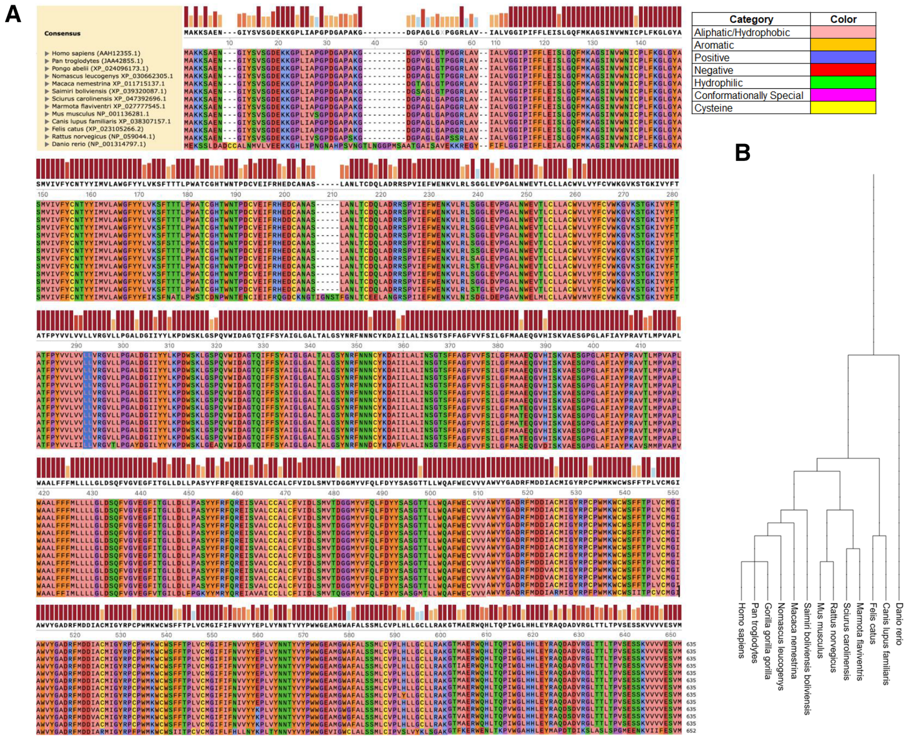

C

Species Name	Identity	Similarity
Pan troglodytes (Chimpanzee)	100%	100%
Gorilla gorilla gorilla (Gorilla)	99.69%	99.84%
Pongo abelii (Orangutan)	99.69%	99.84%
Nomascus leucogenys (Gibbon)	99.69%	99.84%
Macaca nemestrina (Macaque)	99.69%	99.84%
Saimiri boliviensis boliviensis (Squirrel Monkey)	99.53%	99.69%
Sciurus carolinensis (Squirrel)	99.37%	99.53%
Marmota flaviventris (Marmot)	99.21%	99.37%
Mus musculus (Mouse)	98.90%	99.06%
Canis lupus familiaris (Dog)	98.58%	99.37%
Felis catus (Cat)	98.58%	99.37%
Rattus norvegicus (Rat)	98.43%	98.74%
Danio rerio (Zebrafish)	74.25%	83.43%

Figure 1. Sequence alignment between human and other terrestrial mammalian species demonstrates highly similar amino acid sequences of CRT1

(A) Multiple sequence alignment was generated using CLUSTAL Omega, with gap penalties optimized for phylogenetic analysis. (Amino acids 62–108 were excluded from the alignment figure as they were 100% identical.) Analysis includes the amino acid sequence of human, selected terrestrial mammals and zebrafish. Color coding is used to classify each amino acid by type (see legend).

(B) The phylogenetic tree of species studied.

(C) CRT1 identity and similarity compared to human is shown in the table. A change in the transmembrane domain amino acid sequence was exclusive to transmembrane domains 11 or 12.

We report the phylogenetic similarity of the CRT1 amino acid sequence across a group of terrestrial mammals and zebrafish, present an analysis of selected disease-causing *SLC6A8* variants and their impact on creatine transport, and examine how missense variants and location may influence the diagnosis of CRT1 deficiency. These data suggest that the amino acid sequence of CRT1 is highly conserved amongst a select group of terrestrial mammals, that variants commonly occurring in specific TM domains lead to a phenotype from loss of function, and missense mutations in the N- and C-termini are more tolerated. Together, these data provide further understanding into the highly conserved amino acid structure of CRT1, adding insight to the effect of mutations in different regions of this transporter.

Material and methods

SLC6A8 missense mutation analysis

The Leiden Open Variation Database (LOVD) (https://www.lovd.nl/3.0/home) was accessed in March 2025 to analyze reported *SLC6A8* missense variants, both benign/likely benign and pathogenic/likely pathogenic.

Sequence alignment and phylogenetic assessment

CRT1 sequence CLUSTALW alignments[16] were performed with SnapGene (version 7.2) (Dotmatics, GSL Biotech, Boston, MA) with CLUST-Omega analysis to compare sequences. Phylogenetic tree analysis was generated with Interactive Tree of Life (version 7) (https://itol.embl.de/). The accession numbers are in the supplemental information and Figure 1.

SLC6A8 variant synthesis and clinical data

Wild-type (WT) and 9 *SLC6A8* mutations were synthesized as cDNA (Blue Heron Biotech/Eurofins, Bothell, WA); 7 were identified from the Coriell Institute for Medical Research biorepository (https://www.coriell.org/, Camden, NJ) (SD1–7), 1 was provided by a colleague (SD8), and 1 (SD9) was a variant previously used to develop an Slc6a8 murine model (www.jax.org, JAX stock no. 036022). Molecular structure modeling of WT and *SLC6A8* variants was performed using PyMOL Molecular Graphics System (version 3.0, Schrodinger, https://www.pymol.org/). Clinical data were summarized from the Coriell website (SD1–7), communication with the Association for Creatine Deficiencies with a request for information from their institutional review board-approved database, and clinical reports in the case of SD8 and SD9.[17] Each variant cDNA was subcloned into an expression plasmid under the control of the chicken β-actin promoter/cytomegalovirus enhancer.

Competent *Escherichia coli* (One Shot Stbl3, CC737303, Invitrogen, Carlsbad, CA) were individually transformed, amplified in LB media, and underwent plasmid purification by anion-exchange methodology (Invitrogen, K210004).

Determination of CRT1 activity

We transfected 293T cells in DMEM (Thermo Fisher, catalog no. 11885-084, Waltham, MA) with 15% FBS with 0.4588 pmol of plasmid DNA using Lipofectamine 3000 (Invitrogen, catalog no. 3000-015). At 48 h, 100 μM creatine monohydrate (Acros Organics, catalog no. 226795000, Geel, Belgium) in sterile molecular water was added. After 2 h, cells were washed, isolated, and deproteinized (Abcam perchloric acid/KOH deproteinization protocol; Abcam, Waltham, MA). Samples were stored at −80°C until use. Creatine quantification was determined by a colorimetric assay (Abcam, catalog no. ab65339) following the manufacturer's instructions. Samples were run in duplicate with at least 6 biological replicates of each mutation.

MetaDome analysis of missense mutations

MetaDome (https://stuart.radboudumc.nl/metadome/dashboard), using population datasets gnomAD and ClinVar,[18] was accessed in June 2025. This analysis was used to identify regions within *SLC6A8* that are tolerant/intolerant to missense variation, providing a residue-level map to distinguish those that are functionally critical from regions that are more amenable to change. The score interpretation was as follows: <0.5, highly intolerant (i.e., strong purifying selection); 0.5–0.7, moderately intolerant (i.e., some constraint); 0.7–1.0, intermediate tolerance (i.e., moderate functional constraint); and >1.0, tolerant (i.e., variation is well tolerated).

Statistical analysis

Collected data were analyzed with a statistical software package (GraphPad Prism 10 software, San Diego, CA). Results were expressed as mean ± standard deviation. p values were determined using Welch's 1-way ANOVA. $p < 0.05$ was considered significant.

Results

The amino acid sequence of CRT1 is highly conserved in multiple species of terrestrial mammals with identical TM domains 1–10

Alignment was performed assessing similarities in the amino acid sequence of CRT1 in various mammalian species; this was achieved to gain further insight into amino acid differences with implications toward which human mutations may be associated with altered function (Figure 1A). Primate, mouse, rat, dog, and cat, among several other terrestrial mammals, have a highly conserved orthologous amino acid sequence, suggesting this was likely derived through a shared evolutionary history (Figure 1B). While the sequence in humans and chimpanzees is identical, other non-human primate species have high sequence identities (99.69%), each having only 2 different amino acids from humans, typically occurring in the termini, while the squirrel monkey has only 3 amino acid differences (99.53% identity). All of the other mammalian species studied had >98% identity (Figure 1C). The inter-species differences in this highly conserved amino acid sequence were most commonly in the N and C termini or the extracellular/cytoplasmic loops. Amino acid changes in the TM domains were found to be markedly less common; when present, these were limited exclusively to TM11 and TM12. In most circumstances, the amino acid change was within the same class (e.g., branched chain to branched chain, aliphatic to aliphatic [4 of 5]) and less commonly, a change in amino acid class (1 of 5). Comparison of the human sequence with that of the zebrafish (which is 652 amino acids in length), where about 70% of human genes have an ortholog,[19] demonstrated 74.25% identity and 83.43% similarity.

While missense alterations in the CRT1 amino sequence in N and C termini are more likely benign, TM domain missense variants tend to be pathogenic

The LOVD version 3.0 database has multiple mutation categories for *SLC6A8*. As expected, amino acid deletions ($n = 9$) and frameshift or premature stop codon variants ($n = 34$) leading to non-synonymous changes were consistently reported to be pathogenic. Therefore, we focused our mutation analysis on missense mutations (the 20 under clinical classification of benign/likely benign and the 23 classified as pathogenic/likely pathogenic), to determine whether observed consistencies and logical conclusions could be made; these are depicted diagrammatically in Figure 2. Of these 43 mutations, (1) all 8 missense mutations (100%) in the termini (4 in amino, 4 in carboxyl) were classified as benign variants; (2) of the 25 present in the extracellular or cytoplasmic loops, 16 (64%) were classified as pathogenic, while 9 were benign; and (3) of the 10 variants present in TMs, 7 (70%) were classified as pathogenic and 3 as benign. The amino acid sequence from 305 to 415 (including TM 6–8) appears to be a hotspot for missense mutations (see redline demarcation in Figure 2).

MetaDome analysis scoring revealed the missense tolerance of the cytoplasmic termini and TM domains based on homologous domains and missense/synonymous variant frequencies from gnomAD and ClinVar.[18] N termini analysis found a high tolerance to missense variation (score: 1.1–1.4) as did the C termini (score: 0.9–1.3); these data suggest reduced evolutionary constraint in the termini. Conversely, TM1, TM3, TM6, TM8, and TM10 exhibited marked intolerance to missense variants (score: 0.30–0.60), suggesting critical structural and functional roles in creatine binding, gating, or translocation. These domains are highly conserved across *SLC6A8* orthologs. Of the remaining domains, TM4 shows moderate intolerance (0.4–0.7), which may reflect a role in its structural support function; TM7, TM9, and TM12 exhibit moderate tolerance (0.7–1.0); and TM2, TM5, and TM11 exhibit relatively

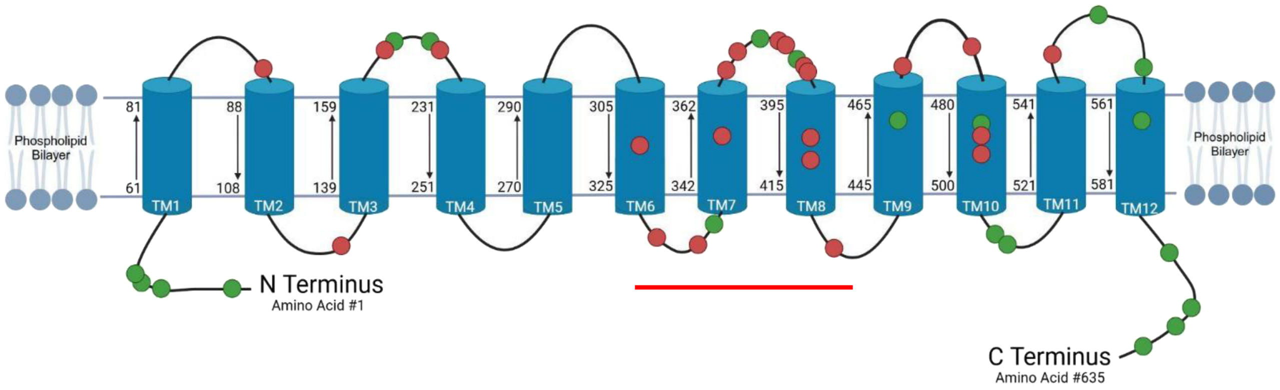

Figure 2. Missense variants of CRT1 and effect on pathogenicity
The linear schematic of CRT1 is populated with 43 missense mutations: 8 in the termini (4 in amino, 4 in carboxyl), 25 in the extracellular or cytoplasmic loops, and 10 in the transmembrane domains. Green dots represent benign/likely benign variants, while red dots represent those that are pathogenic. The amino acid sequence from ~305 to ~415 (represented by the red line) appears to be a hotspot for missense mutations. (In the transmembrane domain, while there are 10 variants, the figure shows only 9 as 2 of the variants affect the same amino acid: Cys491Tyr and Cys491Trp).

high tolerance, suggesting less involvement in substrate transport.

In vitro analysis of disease-associated *SLC6A8* mutations confirms altered creatine transport

To experimentally validate the findings of the analysis, we investigated the functional impact of *SLC6A8* variants on creatine uptake *in vitro*. We functionally studied 9 mutations for their effect on CRT1 function. Each is depicted in its location in a linear schematic of CRT1 (Figure 3A), described by nucleotide and protein location (Figure 3B), and rendered to create a 3-dimensional molecular model (Figure 3C). Of the 9, (1) 1 was a duplication in TM5 (SD3 [yellow]); (2) 3 were amino acid substitutions, 2 in extracellular loops (SD5 [blue] and SD9 [green]) and 1 in TM1 (SD7 [pink]); (3) 1 was a single-nucleotide change resulting in the introduction of a stop codon and a loss of part of an extracellular loop, all of TM12, and the C terminus (SD4 [cyan]); (4) 2 were single amino acid deletions (SD2 [orange], SD8 [black]) in TM8 and TM2; (5) 1 was a large amino acid deletion involving TM10 and TM11 also affecting an intracellular and extracellular loop (SD1 [red]); and (6) 1 was an intronic deletion that led to a frameshift, causing a premature stop codon (SD6 [purple]) with loss of TM6–TM12. Seven of the mutations involved a TM domain(s) and 2 (SD5 and SD9) occurred exclusively in extracellular loops as proline→leucine missense mutations. The clinical phenotypes (Table S1) demonstrated findings consistent with CTD in all (where clinical data were available).

Creatine transport activity was assessed relative to WT. Variants SD1–SD8, which harbored mutations within TM domains or core extracellular loops of CRT1, exhibited marked reductions in creatine uptake, with decreases of ≥88% ($p < 0.0001$ for each) (Figures 4A and 4B). In contrast, SD9, containing a mutation in a peripheral extracellular loop, demonstrated a more modest reduction of 29% ($p = 0.0185$).

Discussion

As the rate of amino acid substitution is determined by structural and functional constraints,[20] the findings of our human variant and species analysis suggest that the CRT1 sequence is essential for its structure and function, with strong negative selection pressure. The amino acid sequences of TMs 1–10 are highly conserved across multiple mammalian species, underscoring their functional importance. The comparative interspecies analysis suggested that in limited circumstances sequence variability in the more peripheral TM domains 11 and 12 may be tolerated without disrupting function. In the case of missense mutations in the N and C termini, the limited data presented here suggest that such variants are more permissible. Furthermore, the analysis demonstrated that mutations can occur anywhere throughout the CRT1 sequence; however, a missense variant hotspot exists from amino acids 305–415, which includes 3 TM domains and 1 cytoplasmic and extracellular loop. In aggregate, these findings highlight the evolutionary conservation of the amino acid sequence of CRT1 across the mammalian species studied and suggest that CRT1 structure and transport activity are highly dependent on a precise amino acid sequence with minimal tolerance for variability. Disruption of this conserved protein architecture, particularly in the core TM domains, leads to significant impairment of function.

Comparative analysis of CRT1 amino acid sequences revealed strong conservation among the terrestrial mammals studied: >99% similarity and >98% identity to the human sequence. This further supports the importance of and the strong evolutionary pressure keeping the CRT1 sequence conserved to maintain function. While some interspecies sequence divergence exists, particularly in the extracellular loops, the core TM domains remain highly conserved. As the closest living relatives of humans,[21] chimpanzees demonstrate >98% identity at

A

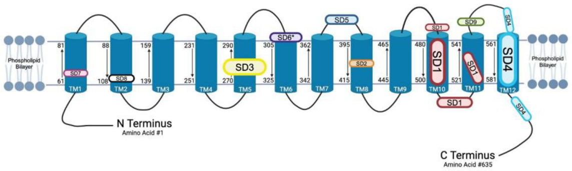

B

	Variant	HGVS cDNA	HGVS Protein	ClinVar ID	Variant Description	Location
SD1	c.1429_1596DEL	NM_005629.4:c.1429_1596del	p.Ser477fs*	RCV000770935	Deletion of nucleotides 1429-1596 (Exons 10-11) in the coding region	Extracellular region before TM10, TM10-TM11, and connecting cytoplasmic region
SD2	p.F408DEL	NM_005629.4:c.1222_1224del	p.Phe408del	VCV000011698.50	Deletion of Phenylalanine in amino acid position 408	TM8
SD3	p.L276_V280DUP	NM_005629.4:c.826_840dup	p.Leu276_Val280dup	VCV000810773.2	Duplication of amino acids 276 (Leucine) – 280 (Valine)	TM5
SD4	p.W556*	NM_005629.4:c.1666G>A	p.Trp556*	Not Found	Nonsense mutation of Tryptophan at amino acid position 556 to a stop codon	Extracellular, TM12, and cytoplasmic regions through the end of the SLC6A8 protein
SD5	p.P382L	NM_005629.4:c.1145C>T	p.Pro382Leu	VCV000533700	Missense mutation of Proline to Leucine at amino acid position 382	Extracellular region between TM7 and TM8
SD6	c.912+36_913-18DEL Intronic Deletion + Premature Stop Codon	NM_005629.4:c.912+36_913-18del	Intronic	Not Found	Deletion in an intronic region resulting in a premature stop codon	TM6 and extracellular and cytoplasmic regions through the end of the SLC6A8 protein
SD7	p.G67D	NM_005629.4:c.199G>A	p.Gly67Asp	Not Found	Missense mutation of Glycine to Aspartic Acid at amino acid position 67	TM1
SD8	p.F107DEL	NM_005629.4:c.318_320delCTT	p.Phe107del	VCV000021448.21	Deletion of Phenylalanine at amino acid position 107	TM2
SD9	p.P544L	NM_005629.4:c.1631C>T	p.Pro544Leu	VCV000065692.9	Missense mutation of Proline to Leucine at amino acid position 544	Extracellular region between TM11 and TM12

C

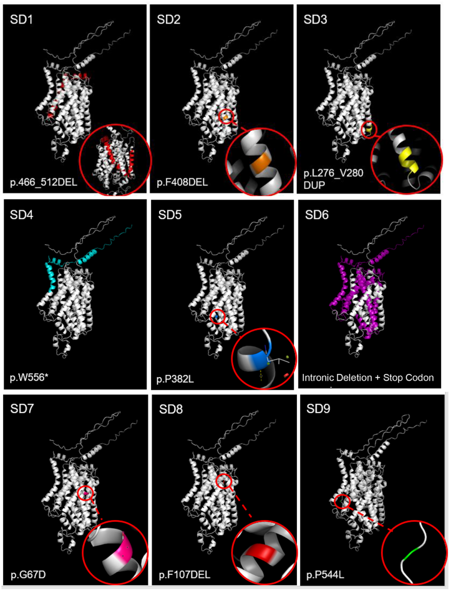

Figure 3. Pathogenic variants of CRT1 and the effect on the amino acid sequence and structure
CRT1 consists of 12 transmembrane domains, extracellular and cytoplasmic loops, and an N and C terminus. Linear schematic of the transporter with the 9 mutation (abbreviated by SD number) locations demonstrated (A), described (B), and represented by 3-dimensional location (C). Colors are coordinated between all subfigures. The asterisk indicates a new mutation (SD6).

nonsynonymous and synonymous DNA nucleotides[22]; therefore it is not surprising that we found the sequence to be identical between the two. A *trans*-species polymorphism at position 38 (V38A) was identified with the alanine variant conserved across all analyzed species except for humans and chimpanzees. This pattern suggest that the valine-to-alanine substitution likely arose more recently, possibly coinciding with the divergence of the human-chimpanzee lineage from the gorilla clade. This finding implies that the sequence has remained unchanged in humans and chimpanzees for the last 6–7 million years.[22]

In addition to the analysis of existing missense variants and mammalian species, the structural characterization of 9 pathogenic variants with integrated creatine uptake data demonstrated transporter dysfunction with severe reduction of cellular creatine uptake in all but 1. Of these, 2 missense mutations were identified with proline-to-leucine variants in extracellular loops: one resulted in markedly decreased creatine transport (SD5, loop between core TM7/TM8), while the other (SD9, loop between TM11/TM12) had only a moderate reduction. The structure of proline facilitates the folding of proteins by the introduction of a rigid turn.[23] We hypothesize that the location of this proline substitution variant in an extracellular loop has a greater effect if in the core of CRT1 than peripherally, where its deleterious effect may be less severe.

In summary, 3 findings emerge from these data. First, the amino acid sequence of CRT1 is highly interspecies conserved between the mammals studied, implying strong negative selection pressure. Second, CRT1 missense mutations may occur at any location, termini, extracellular/cytoplasmic loops, and TM domains; however, a

A

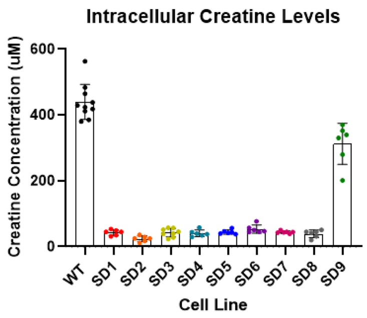

B

Percentage of Intracellular Creatine Uptake Compared to Wild-Type		
Cell Line	Avg [Creatine] (uM)	% of WT
WT	439.8	
SD1	42.9	9.7%
SD2	23.9	5.4%
SD3	35.6	8.1%
SD4	40.3	9.2%
SD5	44.3	10.1%
SD6	53.3	12.1%
SD7	44	10.0%
SD8	38.7	8.8%
SD9	312.3	71.0%

Figure 4. Functional characterization of cellular creatine uptake by pathogenic variants of CRT1
Transmembrane cellular creatine uptake was determined by transfecting individual expression plasmids carrying wild type (WT) of pathogenic mutant *SLC6A8* to 293 cells. The mean uptake of creatine from at least 6 biological replicates is depicted (A), and the percentage of intracellular creatine comparted to WT CRT1 is presented in the table (B). Compared to WT, pathogenic variants SD 1–8 demonstrate a marked reduction (≥88%) of creatine uptake ($p < 0.00001$), while SD9 is less reduced (29%). Data are presented as mean ± standard deviation. Data points are color coordinated with the mutations in Figure 3.

hotspot appears to exist from amino acids 305 to 415. While limited in scope, there was a suggestion that missense variants in core TM domains or cytoplasmic/extracellular loops may be more likely to impact function, while those in the termini tend to be benign. Third, of the variants tested *in vitro* for functional effect, those in the TMs resulted in a marked reduction in creatine transport. *In vitro* findings demonstrating reduced effect of a variant could play a role in determining whether high-concentration oral creatine therapy should be considered in a patient with CTD.

Data and code availability

All data supporting the findings in this study are included in the paper. No custom code was used. Mutation CRT1 plasmids will be provided upon reasonable request to the corresponding author.

Acknowledgments

BioRender scientific image and illustration software (Toronto, Ontario, Canada) was used to create Figures 2 and 3A. These studies were funded by grant no. DISC2-14090 to G.S.L. from the California Institute for Regenerative Medicine.

Declaration of interests

G.S.L. serves as a consultant to Astellas Gene Therapies and has received grant support from the Association of Creatine Deficiencies in an area unrelated to the work described in this paper.

Supplemental information

Supplemental information can be found online at https://doi.org/10.1016/j.xhgg.2025.100489.

Received: April 6, 2025
Accepted: August 1, 2025

References

1. Rosenberg, E.H., Almeida, L.S., Kleefstra, T., deGrauw, R.S., Yntema, H.G., Bahi, N., Moraine, C., Ropers, H.H., Fryns, J. P., deGrauw, T.J., et al. (2004). High prevalence of SLC6A8 deficiency in X-linked mental retardation. Am. J. Hum. Genet. *75*, 97–105. https://doi.org/10.1086/422102.
2. Jangid, N., Surana, P., Salmonos, G., and Jain, V. (2020). Creatine transporter deficiency, an underdiagnosed cause of male intellectual disability. BMJ Case Rep. *13*, e237542. https://doi.org/10.1136/bcr-2020-237542.
3. van de Kamp, J.M., Betsalel, O.T., Mercimek-Mahmutoglu, S., Abulhoul, L., Grünewald, S., Anselm, I., Azzouz, H., Bratkovic, D., de Brouwer, A., Hamel, B., et al. (2013). Phenotype and genotype in 101 males with X-linked creatine transporter deficiency. J. Med. Genet. *50*, 463–472. https://doi.org/10.1136/jmedgenet-2013-101658.
4. van de Kamp, J.M., Mancini, G.M., and Salomons, G.S. (2014). X-linked creatine transporter deficiency: clinical aspects and pathophysiology. J. Inherit. Metab. Dis. *37*, 715–733. https://doi.org/10.1007/s10545-014-9713-8.
5. Mercimek-Andrews, S., and Salomons, G.S. (1993). Creatine Deficiency Disorders. In GeneReviews((R)), M.P. Adam, J. Feldman, G.M. Mirzaa, R.A. Pagon, S.E. Wallace, and A. Amemiya, eds. (University of Washington).
6. Cecil, K.M., Salomons, G.S., Ball, W.S., Jr., Wong, B., Chuck, G., Verhoeven, N.M., Jakobs, C., and DeGrauw, T.J. (2001). Irreversible brain creatine deficiency with elevated serum and urine creatine: a creatine transporter defect? Ann. Neurol. *49*, 401–404. https://doi.org/10.1002/ana.79.
7. Fernandes-Pires, G., and Braissant, O. (2022). Current and potential new treatment strategies for creatine deficiency syndromes. Mol. Genet. Metab. *135*, 15–26. https://doi.org/10.1016/j.ymgme.2021.12.005.
8. Valayannopoulos, V., Boddaert, N., Chabli, A., Barbier, V., Desguerre, I., Philippe, A., Afenjar, A., Mazzuca, M., Cheillan, D., Munnich, A., et al. (2012). Treatment by oral creatine, L-arginine and L-glycine in six severely affected patients with creatine transporter defect. J. Inherit. Metab. Dis. *35*, 151–157. https://doi.org/10.1007/s10545-011-9358-9.
9. Hanna-El-Daher, L., and Braissant, O. (2016). Creatine synthesis and exchanges between brain cells: What can be learned from human creatine deficiencies and various experimental models? Amino Acids *48*, 1877–1895. https://doi.org/10.1007/s00726-016-2189-0.
10. Bai, X., Moraes, T.F., and Reithmeier, R.A.F. (2017). Structural biology of solute carrier (SLC) membrane transport proteins. Mol. Membr. Biol. *34*, 1–32. https://doi.org/10.1080/09687688.2018.1448123.
11. Forrest, L.R., and Rudnick, G. (2009). The rocking bundle: a mechanism for ion-coupled solute flux by symmetrical transporters. Physiology *24*, 377–386. https://doi.org/10.1152/physiol.00030.2009.
12. Farr, C.V., El-Kasaby, A., Freissmuth, M., and Sucic, S. (2020). The Creatine Transporter Unfolded: A Knotty Premise in the Cerebral Creatine Deficiency Syndrome. Front. Synaptic Neurosci. *12*, 588954. https://doi.org/10.3389/fnsyn.2020.588954.
13. Ferrada, E., Wiedmer, T., Wang, W.A., Frommelt, F., Steurer, B., Klimek, C., Lindinger, S., Osthushenrich, T., Garofoli, A., Brocchetti, S., et al. (2024). Experimental and Computational Analysis of Newly Identified Pathogenic Mutations in the Creatine Transporter SLC6A8. J. Mol. Biol. *436*, 168383. https://doi.org/10.1016/j.jmb.2023.168383.
14. Salomons, G.S., van Dooren, S.J., Verhoeven, N.M., Cecil, K. M., Ball, W.S., Degrauw, T.J., and Jakobs, C. (2001). X-linked creatine-transporter gene (SLC6A8) defect: a new creatine-deficiency syndrome. Am. J. Hum. Genet. *68*, 1497–1500. https://doi.org/10.1086/320595.
15. Wyss, M., and Kaddurah-Daouk, R. (2000). Creatine and creatinine metabolism. Physiol. Rev. *80*, 1107–1213. https://doi.org/10.1152/physrev.2000.80.3.1107.
16. Thompson, J.D., Higgins, D.G., and Gibson, T.J. (1994). CLUSTAL W: improving the sensitivity of progressive multiple sequence alignment through sequence weighting, position-specific gap penalties and weight matrix choice. Nucleic Acids Res. *22*, 4673–4680. https://doi.org/10.1093/nar/22.22.4673.
17. Mancini, G.M.S., Catsman-Berrevoets, C.E., de Coo, I.F.M., Aarsen, F.K., Kamphoven, J.H.J., Huijmans, J.G., Duran, M., van der Knaap, M.S., Jakobs, C., and Salomons, G.S. (2005).

Two novel mutations in SLC6A8 cause creatine transporter defect and distinctive X-linked mental retardation in two unrelated Dutch families. Am. J. Med. Genet. *132A*, 288–295. https://doi.org/10.1002/ajmg.a.30473.

18. Wiel, L., Baakman, C., Gilissen, D., Veltman, J.A., Vriend, G., and Gilissen, C. (2019). MetaDome: Pathogenicity analysis of genetic variants through aggregation of homologous human protein domains. Hum. Mutat. *40*, 1030–1038. https://doi.org/10.1002/humu.23798.
19. Howe, K., Clark, M.D., Torroja, C.F., Torrance, J., Berthelot, C., Muffato, M., Collins, J.E., Humphray, S., McLaren, K., Matthews, L., et al. (2013). The zebrafish reference genome sequence and its relationship to the human genome. Nature *496*, 498–503. https://doi.org/10.1038/nature12111.
20. Kimura, M. (1983). The Natural Theory of Molecular Evolution (Cambridge University Press).
21. Suntsova, M.V., and Buzdin, A.A. (2020). Differences between human and chimpanzee genomes and their implications in gene expression, protein functions and biochemical properties of the two species. BMC Genom. *21*, 535. https://doi.org/10.1186/s12864-020-06962-8.
22. Wildman, D.E., Uddin, M., Liu, G., Grossman, L.I., and Goodman, M. (2003). Implications of natural selection in shaping 99.4% nonsynonymous DNA identity between humans and chimpanzees: enlarging genus Homo. Proc. Natl. Acad. Sci. USA *100*, 7181–7188. https://doi.org/10.1073/pnas.1232172100.
23. Melnikov, S., Mailliot, J., Rigger, L., Neuner, S., Shin, B.S., Yusupova, G., Dever, T.E., Micura, R., and Yusupov, M. (2016). Molecular insights into protein synthesis with proline residues. EMBO Rep. *17*, 1776–1784. https://doi.org/10.15252/embr.201642943.

HGGA, Volume *6*

Supplemental information

Evaluation of *SLC6A8* species conservation and the effect of pathogenic variants on creatine transport

Taryn Diep and Gerald S. Lipshutz

Supplementary Table 1

Cell Line	Mutation	Race	Age at First Symptom	Age at Diagnosis	Human Phenotype		Treatment	Notes
SD1	c.1429-1596DEL	Asian	1 year	2.5 years	Global developmental delay<br>Fine motor delay<br>Regression of fine motor skills<br>Delayed social development<br>Abnormal social behavior<br>Short stature<br>Decreased free carnitine<br>Decreased cerebral creatine	HP:0001263<br>HP:0010862<br>HP:0033692<br>HP:0007101<br>HP:0000735<br>HP:0004322<br>HP:0012250<br>HP:0012447	Psychological therapy	Mother screened for elevated TSH and was on thyroid replacement therapy during pregnancy.<br>Mosaic mutation present in 10.38% of cells
SD2	p.F408DEL	White	6 months	1 year	Abnormality of neuronal electrophysiology<br>Encephalopathy<br>Focal-onset seizure<br>Hypotonia<br>Sleep disturbance<br>Delayed speech and language development<br>Global developmental delay<br>Gross motor delay<br>Constipation<br>Feeding difficulties<br>Autism Spectrum Disorder	HP:0012638<br>HP:0001298<br>HP:0007359<br>HP:0001252<br>HP:0002360<br>HP:0000750<br>HP:0001263<br>HP:0002194<br>HP:0002019<br>HP:0011968<br>HP:0000729	N/A	N/A
SD3	p.L276_V280 DUP	White	Birth	10 years	Failure to thrive<br>Global developmental delay<br>Ataxia<br>Autism Spectrum Disorder<br>Intellectual disability<br>Absent speech<br>Abnormal sensory processing<br>Aggressive behavior	HP:0001508<br>HP:0001263<br>HP:0001251<br>HP:0000729<br>HP:0001249<br>HP:0001344<br>HP:0011443<br>HP:0000718	Communication or learning devices; Levetiracetam (Keppra), creatine guconate, arginine base powder, Lorazepam (Ativan).	Family history of hearing problems, learning difficulties, mild depression, and anxiety
SD4	p.W566*	White	1 year	3 years	Global developmental delay<br>Autism Spectrum Disorder	HP:0001263<br>HP:0000729	Speech therapy and communication or learning devices<br>Guanfacine 2ug/day.	MR spectroscopy detected a low creatine peak, high creatine excretion, cerebral creatine deficiency, and increased urinary creatine/creatinine ratio<br>Unaffected carrier mother
SD5	p.P382L	N/A	Under 3 years	6 years	Global developmental delay<br>Failure to thrive<br>Behavioral abnormality<br>Autism Spectrum Disorder<br>Reduced sensitivity to pain<br>Hyperactivity<br>Dystonia<br>Dyskinesia<br>Delayed speech and language development	HP:0001263<br>HP:0001508<br>HP:0000708<br>HP:0000729<br>HP:0007328<br>HP:0000752<br>HP:0001332<br>HP:0100623<br>HP:0000750	N/A	N/A
SD6	c.912+36_913-18DEL Insertion + Stop Codon	N/A	N/A	N/A	N/A	N/A	N/A	Anonymized sample;<br>data unknown
SD7	p.G67D	White	18 months	34 months	Childhood apraxia of speech<br>Delayed speech and language development	HP:0025194<br>HP:0000750	Speech therapy; Arginine, Glycine, Creatine.	EEG was normal, magnetic resonance spectroscopy showed low peak of creatine in brain parenchyma
SD8	p.F107DEL	N/A	2 years	3 months	Febrile seizures<br>Seizure<br>Status epilepticus<br>Focal-onset seizure<br>Drug-resistant epilepsy<br>Behavioral abnormality<br>Intellectual disability	HP:0002373<br>HP:0001250<br>HP:0002133<br>HP:0007359<br>HP:0002128<br>HP:0000708<br>HP:0001249	Stereotactic EEG and right middle frontal gyrus focal resection<br>Glycerol phenylbutrate at 11.2 mL/m2/day	N/A
SD9	p.P544L	White			Psychomotor delay<br>Severe speech and language delay<br>Growth delay<br>Moderate intellectual disability<br>Attention deficit hyperactivity disorder<br>Oral motor apraxia<br>Muscle hypotrophy	HP:0000753<br>HP:0012758<br>HP:0001510<br>HP:0002342<br>HP:0007018<br>HP:0002427<br>HP:0003199	Valproate (no data), methylphenidate	Mother presented with mild learning disabilities, family history negative for developmental or language delay

Supplementary Materials and Methods

Analysis of SLC6A8 Sequences

The accession numbers for the CLUSTALW alignment were: *Homo sapiens* AAH12355.1, *Nomascus leucogenys* (Northern white-cheeked gibbon, an ape species) XP_030662305.1, *Pongo abelii* (Sumatran orangutan) XP_024096173.1, *Gorilla gorilla gorilla* (Western lowland gorilla) XP_030861991.1, *Macaca nemestrina* (Southern pig-tailed macaque, an Old-World monkey) XP_011715137.1, *Pan troglodytes* (Chimpanzee) XP_016803162.2, *Saimiri boliviensis boliviensis* (Black-capped squirrel monkey, a New-World monkey) XP_039320087.1, *Mus musculus* (House mouse) NP_001136281.1, *Sciurus carolinensis* (Eastern gray squirrel) XP_047392696.1, *Rattus norvegicus* (Brown rat) NP_059044.1, *Canis lupus familiaris* (Dog) XP_025316409.1, *Felis catus* (Cat) XP_023105266.2, *Marmota flaviventris* (Yellow-bellied marmot) XP_027777545.1, and *Danio rerio* (zebrafish) NC_133183.1.
